# Genotype and phenotype spectrum of Charcot-Marie-Tooth disease due to mutations in SORD

**DOI:** 10.1093/brain/awaf021

**Published:** 2025-02-13

**Authors:** Andrea Cortese, Maike F Dohrn, Riccardo Curro, Sara Negri, Petra Lassuthova, Chiara Pisciotta, Stefano Tozza, Abdullah Al-Ajmi, Changyong Feng, Pedro J Tomaselli, Gorka Fernandez-Eulate, Saif Haddad, Matilde Laurà, Alexander M Rossor, Elisa Vegezzi, Stefano Facchini, James N Sleigh, Adriana Rebelo, Danique Beijer, Jacquelyn Raposo, Mario Saporta, Barbora Lauerova, Helena F Pernice, Pascal Achenbach, Ulrike Schöne, Tayir Alon, Marcus Deschauer, Isabell Cordts, Carolin D Obermaier, Natalie Winter, Peter D Creigh, Janet E Sowden, Tyler Rehbein, Stefania Magri, Alessandro Bertini, Paola Saveri, Paolo Ripellino, Jingyu Huang, Aleksandra Nadaj-Pakleza, Alison Ross, James K L Holt, Kathryn M Brennan, Rivka Sukenik-Halevy, Varoona Bizaoui, Yesim Parman, Esra Battaloglu, Arman Cakar, Hadil Alrohaif, Simon Hammans, Kishore R Kumar, Marina L Kennerson, Hülya Kayserili, Defne A Amado, Katrin Hahn, Paola Valentino, Francesca Cavalcanti, Carlo Gaetano, Franco Taroni, Geir J Braathen, Henry Houlden, Tanya Stojkovic, Stojan Peric, Alessandra Bolino, Stefano C Previtali, Lee Yi-Chung, Ayşe N Başak, Sherifa A Hamed, Ricardo Rojas-Garcia, Kristl G Claeys, Wilson Marques, Teresa Sevilla, Beate Schlotter-Weigel, Fiore Manganelli, Ruxu Zhang, David N Herrmann, Steven S Scherer, Pavel Seeman, Davide Pareyson, Mary M Reilly, Michael E Shy, Stephan Züchner

**Affiliations:** Department of Neuromuscular Diseases, UCL Queen Square Institute of Neurology, London WC1N 3BG, UK; Department of Brain and Behavioral Sciences, University of Pavia, Pavia 27100, Italy; Dr. John T. Macdonald Foundation, John P. Hussman Institute for Human Genomics, University of Miami, Miami, FL 33136, USA; Department of Neurology, Medical Faculty, RWTH Aachen University, Aachen 52074, Germany; Department of Neuromuscular Diseases, UCL Queen Square Institute of Neurology, London WC1N 3BG, UK; Department of Brain and Behavioral Sciences, University of Pavia, Pavia 27100, Italy; Laboratorio di Epigenetica, Dipartimento Medicina Riabilitativa NeuroMotoria—MeRiNM, Istituti Clinici Scientifici Maugeri IRCCS, Pavia 27100, Italy; Department of Paediatric Neurology, 2nd Faculty of Medicine, Charles University in Prague and Motol University Hospital, Praha 150 06, Czechia; Unit of Rare Neurological Diseases, Department of Clinical Neurosciences, Fondazione IRCCS Istituto Neurologico Carlo Besta, Milan, 20133, Italy; Department of Neurosciences, Reproductive Sciences and Odontostomatology, University of Naples Federico II, Naples 80131, Italy; Division of Neurology, Department of Medicine, Al-Jahra Hospital, Al-Jahra 00020, Kuwait; Department of Biostatistics and Computational Biology, University of Rochester, Rochester, NY 14642, USA; Clinical Hospital of Ribeirão Preto, Department of Neurosciences and Behaviour Sciences, University of São Paulo, Ribeirão Preto 14015-010, Brazil; Nord/Est/Ile-de-France Neuromuscular Diseases Reference Center, Pitié-Salpêtrière Hospital, APHP, Paris 75013, France; Department of Neuromuscular Diseases, UCL Queen Square Institute of Neurology, London WC1N 3BG, UK; Department of Neuromuscular Diseases, UCL Queen Square Institute of Neurology, London WC1N 3BG, UK; Department of Neuromuscular Diseases, UCL Queen Square Institute of Neurology, London WC1N 3BG, UK; IRCCS Mondino Foundation, Pavia 27100, Italy; Department of Neuromuscular Diseases, UCL Queen Square Institute of Neurology, London WC1N 3BG, UK; Department of Brain and Behavioral Sciences, University of Pavia, Pavia 27100, Italy; Department of Neuromuscular Diseases, UCL Queen Square Institute of Neurology, London WC1N 3BG, UK; UK Dementia Research Institute, University College London, London WC1E 6BT, UK; Dr. John T. Macdonald Foundation, John P. Hussman Institute for Human Genomics, University of Miami, Miami, FL 33136, USA; Dr. John T. Macdonald Foundation, John P. Hussman Institute for Human Genomics, University of Miami, Miami, FL 33136, USA; Translational Genomics of Neurodegenerative Diseases, Hertie-Institute for Clinical Brain Research, University of Tübingen, Tübingen 72076, Germany; Dr. John T. Macdonald Foundation, John P. Hussman Institute for Human Genomics, University of Miami, Miami, FL 33136, USA; Dr. John T. Macdonald Foundation, John P. Hussman Institute for Human Genomics, University of Miami, Miami, FL 33136, USA; Department of Paediatric Neurology, 2nd Faculty of Medicine, Charles University in Prague and Motol University Hospital, Praha 150 06, Czechia; Charité University Medicine Berlin, Corporate Member of Freie Universität Berlin and Humboldt-Universität Zu Berlin, Department of Neurology and Experimental Neurology, Berlin 10117, Germany; Department of Neurology, Medical Faculty, RWTH Aachen University, Aachen 52074, Germany; Institute of Neuropathology, Medical Faculty, RWTH Aachen University, Aachen 52074, Germany; Department of Neurology, Medical Faculty, RWTH Aachen University, Aachen 52074, Germany; Department of Neurology, Maria Hilf Hospital Mönchengladbach, Mönchengladbach 41063, Germany; Department of Neurology, Beilinson Hospital, Rabin Medical Center, Petah Tikva 4941492, Israel; Department of Neurology, University Hospital rechts der Isar, School of Medicine and Health, Technical University Munich, München 81675, Germany; Department of Neurology, University Hospital rechts der Isar, School of Medicine and Health, Technical University Munich, München 81675, Germany; Center for Genomics and Transcriptomics Tübingen and Zentrum für Humangenetik Tübingen, Tübingen 72076, Germany; Department of Neurology and Hertie Institute for Clinical Brain Research (HIH), University of Tübingen, Tübingen 72076, Germany; Department of Neurology, University of Rochester, Rochester, NY 14642, USA; Department of Neurology, University of Rochester, Rochester, NY 14642, USA; Department of Neurology, University of Rochester, Rochester, NY 14642, USA; Unit of Medical Genetics and Neurogenetics, Fondazione IRCCS Istituto Neurologico Carlo Besta, Milan 20133, Italy; Unit of Rare Neurological Diseases, Department of Clinical Neurosciences, Fondazione IRCCS Istituto Neurologico Carlo Besta, Milan, 20133, Italy; Unit of Rare Neurological Diseases, Department of Clinical Neurosciences, Fondazione IRCCS Istituto Neurologico Carlo Besta, Milan, 20133, Italy; Department of Neurology, Neurocenter of Southern Switzerland EOC, Ospedale Regionale di Lugano, Lugano 6900, Switzerland; Faculty of Biomedical Sciences, Università della Svizzera Italiana, Lugano 6900, Switzerland; Dr. John T. Macdonald Foundation, John P. Hussman Institute for Human Genomics, University of Miami, Miami, FL 33136, USA; Service de neurologie, Centre de référence des maladies neuromusculaires, Hôpitaux Universitaires de Strasbourg, Strasbourg 67091, France; Clinical Genetics, NHS Grampian, Aberdeen AB15 6RE, UK; Department of Neurology, The Walton Centre, Liverpool L9 7LJ, UK; Institute of Neurological Sciences, Queen Elizabeth University Hospital, Glasgow G51 4TF, UK; Raphael Recanati Genetic Institute, Rabin Medical Center—Beilinson Hospital, Petah Tikva 4941492, Israel; School of Medicine, Faculty of Medical and Health Sciences, Tel Aviv University, Tel Aviv 6997801, Israel; Service de Génétique, Centre Hospitalier Universitaire Caen Normandie, Caen 14000, France; Neuromuscular Unit, Department of Neurology, Istanbul University, Istanbul Faculty of Medicine, Istanbul 1827, Turkey; Department of Molecular Biology and Genetics, Center for Life Sciences and Technologies, Bogazici University, Istanbul 34342, Turkey; Neuromuscular Unit, Neurology Department, Istanbul Faculty of Medicine, Istanbul University, Istanbul 34093, Turkey; Kuwait Medical Genetics Centre, Sabah Hospital, Kuwait City, Kuwait; Wessex Neurological Centre, University Hospital Southampton NHS Foundation Trust. Southampton, SO16 6YD, UK; Molecular Medicine Laboratory and Department of Neurology, Concord Repatriation General Hospital, Concord Clinical School, The University of Sydney, Concord, NSW 2006, Australia; Translational Neurogenomics Group, Genomic and Inherited Disease Program, Garvan Institute of Medical Research, Sydney, NSW 2010, Australia; St Vincent's Healthcare Clinical Campus, UNSW Medicine and Health, UNSW Sydney, Kensington, NSW 2052, Australia; Northcott Neuroscience Laboratory, ANZAC Research Institute, Sydney Local Health District, Concord and School of Medical Sciences, Faculty of Medicine and Health, University of Sydney, Sydney, NSW 2050, Australia; Department of Medical Genetics, Koc University School of Medicine (KUSOM), Istanbul 34010, Turkey; Department of Neurology, The Perelman School of Medicine at the University of Pennsylvania, Philadelphia, PA 19104, USA; Charité University Medicine Berlin, Corporate Member of Freie Universität Berlin and Humboldt-Universität Zu Berlin, Department of Neurology and Experimental Neurology, Berlin 10117, Germany; Department of Medical and Surgical Sciences, Institute of Neurology, University Magna Graecia, Catanzaro 88100, Italy; Institute for Biomedical Research and Innovation (IRIB), Department of Biomedical Sciences, National Research Council (CNR), Mangone (CS) 87050, Italy; Laboratorio di Epigenetica, Dipartimento Medicina Riabilitativa NeuroMotoria—MeRiNM, Istituti Clinici Scientifici Maugeri IRCCS, Pavia 27100, Italy; Unit of Medical Genetics and Neurogenetics, Fondazione IRCCS Istituto Neurologico Carlo Besta, Milan 20133, Italy; Department of Medical Genetics, Telemark Hospital Trust, Skien 3710, Norway; Department of Neuromuscular Diseases, UCL Queen Square Institute of Neurology, London WC1N 3BG, UK; Nord/Est/Ile-de-France Neuromuscular Diseases Reference Center, Pitié-Salpêtrière Hospital, APHP, Paris 75013, France; Neurology Clinic, University Clinical Center of Serbia, Faculty of Medicine, University of Belgrade, Belgrade 11000, Serbia; Human Inherited Neuropathies Unit, Division of Neuroscience, Institute of Experimental Neurology, IRCCS Ospedale San Raffaele, Milan 20132, Italy; Vita-Salute San Raffaele University, Milan 20132, Italy; Neuromuscular Repair Unit, Division of Neuroscience, IRCCS Ospedale San Raffaele, Milan 20132, Italy; Department of Neurology, Taipei Veterans General Hospital, Taipei 11217, Taiwan; Brain Research Center, National Yang Ming Chiao Tung University, Taipei 112304, Taiwan; Koç University, School of Medicine, Suna and İnan Kıraç Foundation, Neurodegeneration Research Laboratory (NDAL), Research Center for Translational Medicine, Istanbul 34010, Turkey; Department of Neurology and Psychiatry, Assiut University Hospital, Assiut 71515, Egypt; Department of Neurology, Neuromuscular Diseases Unit, Hospital de la Santa Creu i Sant Pau, Universitat Autònoma de Barcelona, Barcelona 08193, Spain; Center for Networked Biomedical Research into Rare Diseases (CIBERER), Madrid 28029, Spain; Department of Neurology, University Hospitals Leuven, Leuven 3000, Belgium; Laboratory for Muscle Diseases and Neuropathies, Department of Neurosciences, KU Leuven, and Leuven Brain Institute (LBI), Leuven 3000, Belgium; Clinical Hospital of Ribeirão Preto, Department of Neurosciences and Behaviour Sciences, University of São Paulo, Ribeirão Preto 14015-010, Brazil; Neurology Department, La Fe Health Research Institute (IISLAFE), Neuromuscular Research Unit, Valencia 46026, Spain; Friedrich Baur Institute at the Department of Neurology, LMU University Hospital, LMU Munich, München 80336, Germany; Department of Neurosciences, Reproductive Sciences and Odontostomatology, University of Naples Federico II, Naples 80131, Italy; Department of Neurology, Third Xiangya Hospital, Central South University, Changsha 410013, China; Department of Neurology, University of Rochester, Rochester, NY 14642, USA; Department of Neurology, The Perelman School of Medicine at the University of Pennsylvania, Philadelphia, PA 19104, USA; Department of Paediatric Neurology, 2nd Faculty of Medicine, Charles University in Prague and Motol University Hospital, Praha 150 06, Czechia; Unit of Rare Neurological Diseases, Department of Clinical Neurosciences, Fondazione IRCCS Istituto Neurologico Carlo Besta, Milan, 20133, Italy; Department of Neuromuscular Diseases, UCL Queen Square Institute of Neurology, London WC1N 3BG, UK; Department of Neurology, Carver College of Medicine, University of Iowa, Iowa City, IA 52242, USA; Dr. John T. Macdonald Foundation, John P. Hussman Institute for Human Genomics, University of Miami, Miami, FL 33136, USA

**Keywords:** SORD, natural history, hereditary neuropathy, polyol pathway, aldose reductase

## Abstract

Biallelic loss-of-function mutations in the sorbitol dehydrogenase (*SORD*) gene cause the most common recessive type of Charcot-Marie-Tooth disease (CMT), CMT-SORD. However, the full genotype-phenotype spectrum and progression of the disease remain to be defined. Notably, a multicentre phase 2/3 study to test the efficacy of govorestat (NCT05397665), a new aldose reductase inhibitor, is currently ongoing. Diagnosing CMT-SORD will become imperative when disease-modifying therapies become available.

In this cross-sectional multicentre study, we identified 144 patients from 126 families, including 99 males (69%) and 45 females (31%). Patients represented multiple ancestries, including European, Hispanic, Chinese, Near Eastern and Northern African. We confirmed c.757delG (p.Ala253GlnfsTer27) as the most common pathogenic allele, followed by c.458C>A (p.Ala153Asp), while other variants were identified, mostly in single cases. The average sorbitol level in CMT-SORD patients was significantly higher compared to controls and heterozygous carriers, independently from serum storage duration, sex or variant type. Two-thirds of cases were diagnosed with CMT2 while one-third had distal hereditary motor neuropathy. Disease onset was usually in the second decade of life. Although foot dorsiflexion was the most affected muscle group, dorsal and plantar flexion had a similar degree of weakness in most cases (difference of Medical Research Council score ≤ 1). One-fourth of patients used ankle foot orthoses, usually in their 30s, but most patients maintained independent ambulation later in life. Nerve conduction studies were suggestive of a motor predominant axonal neuropathy, with reduced conduction velocities in the intermediate range in a quarter of the cases. Sensory conductions in the upper limbs appeared more frequently affected than in the lower limbs. Foot dorsiflexion and plantar flexion decreased significantly with age. Male sex was significantly associated with the severity of distal lower limb weakness (plantar flexion) and a larger change over time (dorsiflexion).

In conclusion, CMT-SORD is a frequent recessive form of axonal, motor predominant CMT, with prominent foot dorsiflexion and plantar flexion involvement. Fasting serum sorbitol is a reliable biomarker of the condition that can be utilized for pathogenicity assessment of identified rare *SORD* variants.

## Teaser

Mutations in sorbitol dehydrogenase cause the most common recessive form of Charcot-Marie-Tooth disease, CMT-SORD. By enrolling 144 patients, Cortese *et al.* characterize the full genotype and phenotype spectrum of CMT-SORD, providing information necessary to support early diagnosis and accurate tracking of disease progression.

## Introduction

Hereditary neuropathies comprise a broad group of over 100 different, genetically defined diseases with a wide genotype-phenotype spectrum. The term Charcot-Marie-Tooth disease (CMT) is increasingly used as an umbrella term for non-syndromic inherited neuropathies that affect sensory and motor axons. We recently identified biallelic mutations in the *SORD* gene, encoding sorbitol dehydrogenase, as a cause of hereditary motor neuropathy and hereditary motor and sensory neuropathy, here referred to as CMT-SORD.^1^ Based on the allele frequency of the most common c.757delG (p.Ala253GlnfsTer27) mutation (∼0.3% of all chromosomes) across many populations according to GnomAD, we calculated a prevalence of at least 3000 CMT-SORD cases in the USA alone, making CMT-SORD as the likely most common recessive form of CMT. Indeed, the high frequency of CMT-SORD has been confirmed by several independent studies and across different ethnicities (OMIM phenotype number = 618912).^1-12^

CMT-SORD affects the well known polyol pathway,^13^ which facilitates the conversion of glucose to fructose in two steps—generating sorbitol through the enzyme aldose reductase (AR) and then converting sorbitol to fructose via SORD. This process has been broadly investigated in the context of diabetic neuropathies.^14-16^ Biallelic pathogenic *SORD* mutations result in a loss of SORD function and lead to a conspicuous accumulation of sorbitol in patient serum and fibroblasts.^1^ A promising clinical trial with a novel AR inhibitor, AT-007/govorestat,^17^ is ongoing (NCT05397665), motivating a further characterization of the full clinical and biochemical phenotypic and genotypic spectrum of CMT-SORD. Herein, we report a cross-sectional observation of 144 CMT-SORD patients and their pathogenic alleles, including frequencies and associated phenotypic variation. We also confirm the reliability of sorbitol as a biomarker of the disease.

## Materials and methods

### Patients

Patients were examined by experienced neurologists at different Neuromuscular Reference Centres. The study design conformed to the Declaration of Helsinki, and ethical approval was obtained at each site prior to study initiation. For inclusion, patients had to carry bi-allelic mutations in the *SORD* gene or to have high serum sorbitol levels if segregation of variants was not possible. We collected detailed information on patient history, using a standardized protocol distributed to all sites. An initial, full neurologic examination and, when available, a second evaluation, were obtained. When recorded, disease severity was scored using the previously validated Charcot-Marie-Tooth Examination Score (CMTESv2).^18^

### Nerve conduction studies

Previously conducted nerve conduction results were re-assessed. We collected original values of compound motor action potentials (CMAP), motor nerve conduction velocity (NCV), distal motor latency, and F-waves from the median, ulnar, tibial and peroneal nerves. Sensory nerve action potentials (SNAPs) and sensory NCV were measured (orthodromically or antidromically) at median, ulnar, radial and sural nerves. Patients were labelled CMT2 if both motor and sensory nerves were affected, or distal hereditary motor neuropathy (dHMN) if the neuropathy affected motor but not sensory axons.

### Molecular genetic analyses

Patients were diagnosed at multiple sites, with genetic analyses being performed in different certified genetic laboratories.^19^ Either whole-genome sequencing, whole-exome sequencing, targeted gene panels, or Sanger sequencing were performed, as described in Cortese *et al*.^1^

### Sorbitol measurements

Serum samples were obtained in 30 patients following a fasting period of at least 8 h. In the reference laboratory, samples were measured using liquid-chromatography mass spectrometry.^1^

### Data evaluation and statistics

For continuous variables, mean values and standard deviations (SD) were reported, and the normality assumption of their distribution was checked using the Shapiro-Wilk test. Two-sample *t*-tests were employed for normally distributed data to compare mean values between the two groups, while paired *t*-tests were used to examine changes within the same group. For skewed data, the corresponding Wilcoxon rank sum test (or signed rank test) was utilized. Categorical variables were analysed using Pearson's chi-square test or Fisher's exact test to compare distributions between the two groups. Multiple linear regression was conducted to investigate the association between each primary outcome variable and demographic and clinical covariates. The significance level was set at 0.05 for all analyses. The analyses were performed using SAS 14 (SAS Institute Inc., Cary, North Carolina, USA). Graphs were generated using GraphPad Prism version 9.4.1 for Windows (GraphPad Software, San Diego, California, USA).

## Results

### Genotype spectrum of CMT-SORD

**Figure 1:**
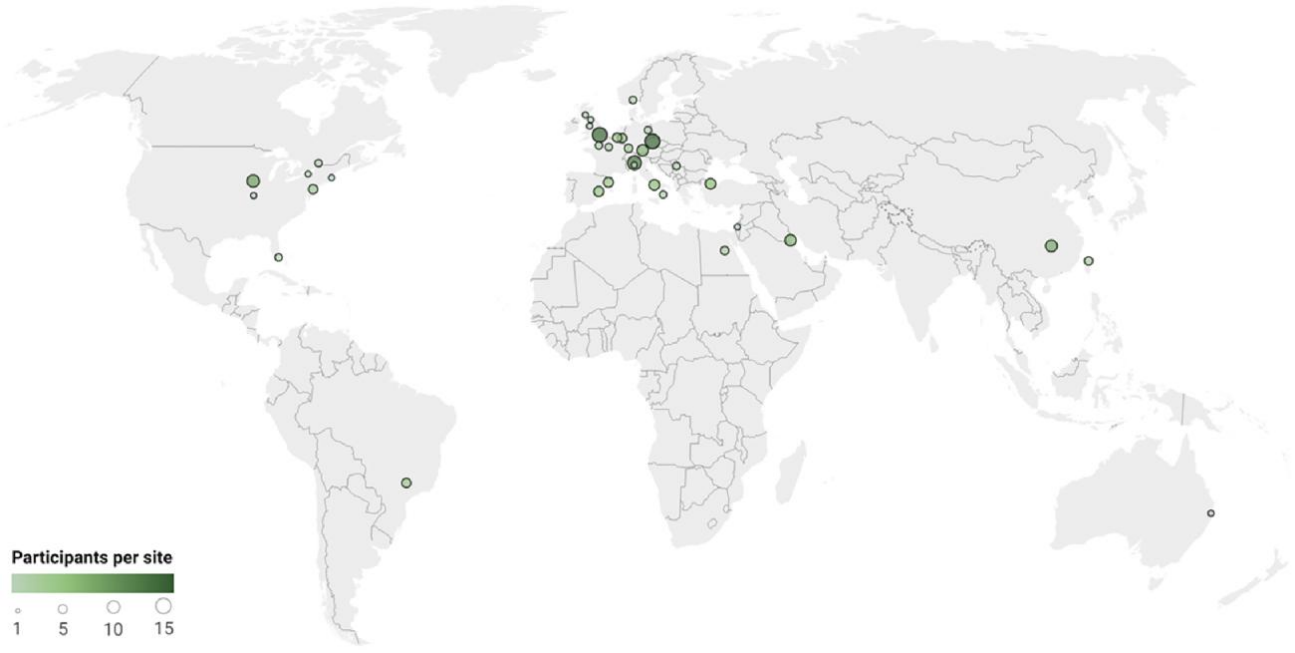
**Geographic distribution of recruited patients with CMT-SORD**.

**Figure 2:**
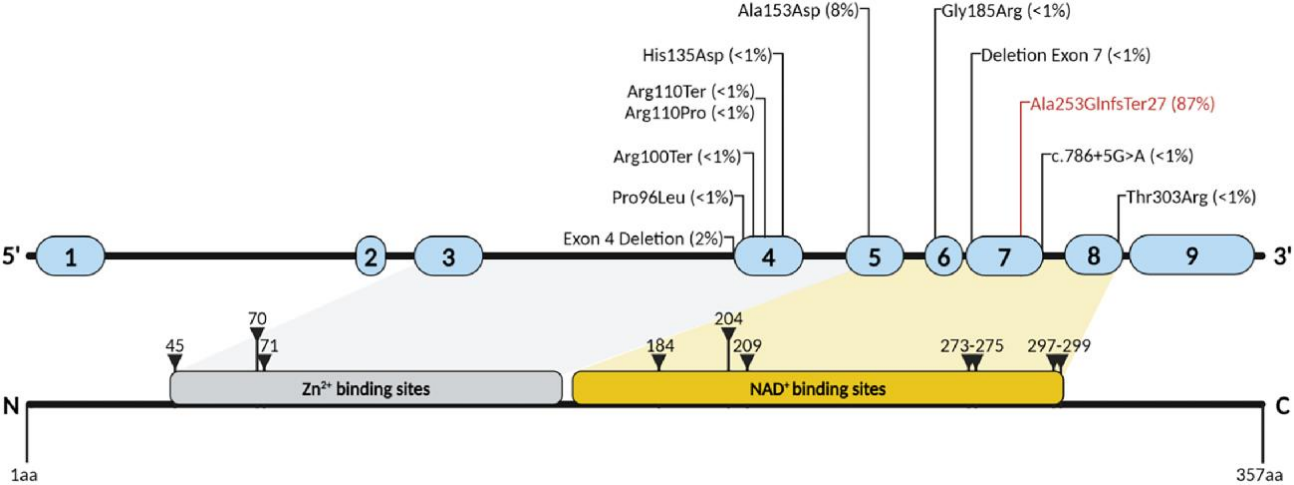
***SORD* variants detected in this study.** Linear depiction of the *SORD* gene and the corresponding sorbitol dehydrogenase monomer domains. The most frequent frameshift mutation is shown in red. Amino acid positions for binding sites were derived from UniProt.

We identified 144 patients from 126 families and 43 centres carrying biallelic mutations in *SORD* (Fig. 1). There were 99 males (69%) and 45 females (31%). Average age at study enrolment was 40.9 ± 14.8 years (range 15–75). Forty-seven (33%) patients had a family history of neuropathy and 26 (18%) were born from consanguineous parents, including eight individuals with an additional affected family member. Thus, in 79 (55%) individuals the disease was sporadic, without report of family history of neuropathy or consanguinity. We confirmed c.757delG (p.Ala253GlnfsTer27) as the most common pathogenic allele, followed by c.458C>A (p.Ala153Asp), while the other variants were identified mostly in single cases (Table 1 and Fig. 2). Altogether, 113 (78%) patients carried a homozygous c.757delG (p.Ala253GlnfsTer27) variant, 25 (18%) were compound heterozygous for the c.757delG (p.Ala253GlnfsTer27) and a second nonsense, splicing, exon deletion or missense variant, while only six (4%) individuals carried two different variants from c.757delG (p.Ala253GlnfsTer27). Overall, 118 (82%) had biallelic nonsense changes, including splicing and structural variants, while 26 (18%) had at least one missense variant. In 17 patients, carrying two heterozygous mutations, testing of additional family members provided evidence that the two mutations were located on separate alleles. In 11 patients for whom segregation was not possible, the compound heterozygous state could be inferred from the detection of a high serum sorbitol level in the pathogenic range.

**Table 1:** Genotype spectrum of CMT-SORD

Patients	Cases	Alleles	%
Allele 1/allele 2			
c.757delG (p.Ala253GlnfsTer27)/c.757delG (p.Ala253GlnfsTer27)	113	–	78%
c.757delG (p.Ala253GlnfsTer27)/c.458C>A (p.Ala153Asp)	17	–	12%
c.757delG (p.Ala253GlnfsTer27)/other mutation	8	–	6%
c.458C>A (p.Ala153Asp)/c.458C>A (p.Ala153Asp)	2	–	1%
c.458C>A (p.Ala153Asp)/other mutation	2	–	1%
Other mutation/other mutation	2	–	1%
**Alleles**			
c.757delG (p.Ala253GlnfsTer27)	–	251	87%
c.458C>A (p.Ala153Asp)	–	23	8%
Exon 4 deletion	–	3	2%
c.786+5G>A; p.?	–	2	<1%
c.908C>G (p.Thr303Arg)	–	2	<1%
c.287C>T (p. Pro96Leu)	–	1	<1%
c.298C>T (p.Arg100Ter)	–	1	<1%
c.328C>T (p.Arg110Ter)	–	1	<1%
c.329G>C (p.Arg110Pro)	–	1	<1%
c.403C>G (p.His135Asp)	–	1	<1%
c.553G>A (p.Gly185Arg)	–	1	<1%
Exon 7 deletion	–	1	<1%

### Serum sorbitol level is a reliable biomarker of CMT-SORD

**Figure 3:**
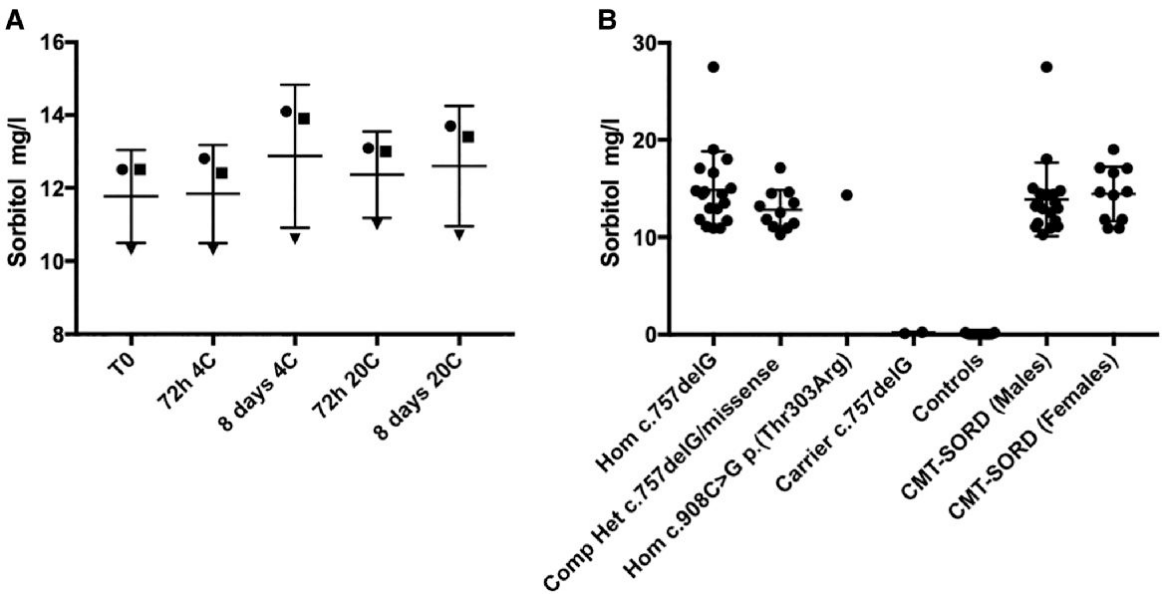
**Fasting serum sorbitol level in CMT-SORD.** (**A**) Stability of sorbitol metabolite in serum kept at 4°C or at room temperature for 3 or 8 days. (**B**) Fasting serum sorbitol level in CMT-SORD patients, *SORD* mutation carriers and controls according to variant type.

To test the stability of sorbitol in sera over time and at different temperatures, we performed a time series using serum samples from three patients. Serum sorbitol proved to be stable as there was no significant difference in the level measured immediately after thawing of snap frozen sera or on samples kept refrigerated at 4° or at room temperature for either 72 h or 8 days (Fig. 3A). These observations facilitated collection and testing of sera from multiple centres worldwide, as sera could be collected and shipped at room temperature for testing. Serum sorbitol levels were available in 30 cases, including 18 cases carrying biallelic nonsense or splicing variants, 11 individuals carrying one nonsense or splicing variant and one missense variant, and one case carrying a homozygous c.908C>G (p.Thr303Arg) missense variant. The average sorbitol level in CMT-SORD patients was 14.7 ± 4.9 mg/l (range 10.3–27.0), which was significantly higher compared to controls (0.07 ± 0.06, *P* < 0.001) and to carriers of one heterozygous nonsense variant (Fig. 3B). We found no significant difference between sorbitol level in males versus females (13.9 ± 0.9 versus 14.4 ± 0.8, *P* = 0.67). Also, we did not detect significant fluctuations of serum sorbitol after overnight fasting and 1 h or 3 h after a meal (Supplementary Fig. 1). Increased serum sorbitol levels provided evidence of pathogenicity for six missense variants c.287C>T (p.Pro96Leu; novel), c.329G>C (p.Arg110Pro), c.403C>G (p.His135Asp), c.458C>A (p.Ala153Asp), c.553G>A (p.Gly185Arg), c.908C>G (p.Thr303Arg; novel), and one splicing variant c.786+5G>A; p.?. Sorbitol levels further confirmed the pathogenicity of two structural variants causing exon deletion (Fig. 2 and Table 1). Furthermore, this biochemical test provided indirect evidence of the in-trans allelic status of heterozygous variants in cases where segregation of variants through additional family members was not possible. Importantly, there was no association between serum sorbitol levels and age at sorbitol test, sex, or missense versus nonsense variants.

### Clinical features of patients with CMT-SORD

Two-thirds of cases were diagnosed with CMT2 while one-third had dHMN (Table 2). There was no association between CMT subtype and mutation type (nonsense versus missense). Disease onset was usually in the second decade of life, however, 79% of patients reported foot deformities and 46% described poor performance in sport activities at school, suggesting an earlier onset of disease. Nonetheless, motor developmental delay and scoliosis were rare, which is different from most recessive CMT forms. Interestingly, a high proportion of patients reported difficulties standing on both their toes and heels, starting at ∼18 years, suggesting an early involvement of foot plantar flexion, as in other forms of dHMN. Also, 28% of patients reported distal tremor of the upper limbs, which is also not uncommon in motor predominant neuropathies. Approximately 37% of individuals reported a reduced hand dexterity, on average 8 years after the onset of walking difficulties. Reduced sensation and paraesthesia were reported by less than a third of patients, and neuropathic pain was uncommon. One out of four patients used ankle foot orthoses, usually in their 30s, however only 13 patients needed a stick/cane, and two used a wheelchair. A neurologic comorbidity was reported in six patients, including epilepsy, multiple sclerosis, subarachnoid haemorrhage, intellectual disability, stroke and Kennedy disease, which were all considered unlikely to be related to SORD mutations. Four patients had type 2 diabetes mellitus, and none had cataracts.

**Table 2:** Clinical characteristics of patients diagnosed with CMT-SORD

	*n* (%)	Mean ± SD (min-max), *n*^a^
Male	99 (69%)	–
Age at study enrolment	–	40.9 ± 14.8 (15.0–74.8)
Disease duration (since onset of walking difficulties)	–	23.0 ± 13.9 (1.5–61.9), *n* = 102
Family history of neuropathy	–	–
Sporadic	79 (55%)	–
Affected family member	47 (33%)	–
Consanguinity	26 (18%)	–
Consanguinity and affected family member	8 (6%)	–
Ethnicity	–	–
European	108 (75%)	–
Middle Eastern	16 (11%)	–
East-Asian	13 (9%)	–
Black	3 (2%)	–
Hispanic	2 (1%)	–
Unknown	2 (1%)	–
CMT subtype	**–**	–
CMT2	86 (60%)	–
dHMN	58 (40%)	–
Motor delay	7 (5%)	–
Foot deformities	114 (79%)	–
Foot surgery	13 (9%)	–
Hand surgery	3 (2%)	–
Scoliosis	22 (15%)	–
Difficulties with sport in school	66 (46%)	–
Sensory loss	45 (31%)	–
Paraesthesia	36 (25%)	–
Cramps	36 (25%)	–
Neuropathic pain	23 (16%)	–
Need for pain medication	11 (8%)	–
Difficulties running	127 (88%)	15.5 ± 7.8 (4–47) *n* = 85
Difficulties walking	123 (85%)	17.5 ± 8.9 (3–50) *n* = 102
Difficulties standing on the heels	115 (80%)	17.8 ± 8.8 (7–50) *n* = 57
Difficulties standing on the toes	96 (67%)	18.9 ± 9.7 (7.00–57) *n* = 53
Impaired hand dexterity	53 (37%)	26.3 ± 12.9 (12–62) *n* = 34
Distal upper limb tremor	40 (28%)	21.8 ± 11.0 (10–52) *n* = 21
Use of walking aids	–	–
Insoles	33 (23%)	24.1 ± 10.8 (9–45) *n* = 23
AFOs	40 (28%)	34.1 ± 13.0 (13–65) *n* = 26
stick/cane	13 (9%)	47.2 ± 24.3 (27–67) *n* = 6
wheelchair	2 (1%)	NA

AFOs = ankle-foot orthoses; dHMN = distal hereditary motor neuropathy; NA = not available; SD = standard deviation.

^a^Age at symptom onset, Number of individuals (if different from 144).

### Neurological examination

**Figure 4:**
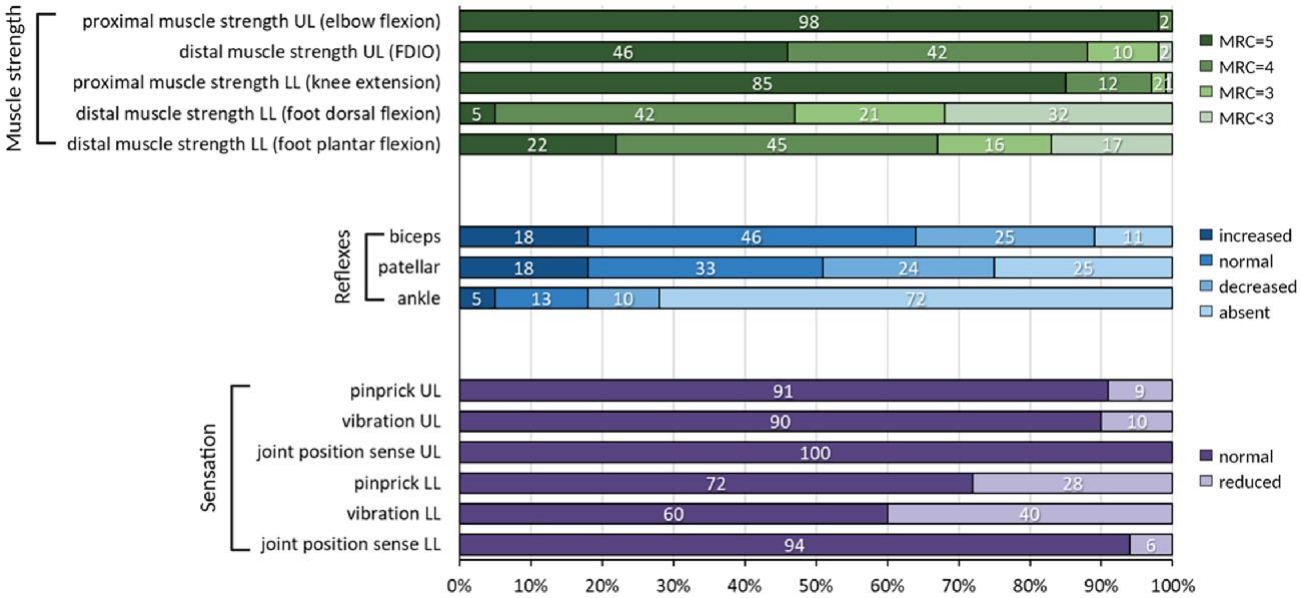
**Neurologic findings in patients with CMT-SORD.** Number of individuals = 139; disease duration = 15.4 ± 11.3 years since the onset of walking difficulties. FDIO = first dorsal interosseous muscle; LL = lower limbs; MRC = Medical Research Council; UL = upper limbs.

A detailed neurological assessment was available in 139 cases at an average age of 33.7 ± 13.8 years (Fig. 4). Distal atrophy was observed in 98% of cases. Muscle strength was usually normal in the proximal muscle groups of the upper and lower limbs. Half of the patients had reduced strength of intrinsic hand muscles, while most patients had reduced strength of distal lower limb muscles. Foot dorsiflexion was the most affected, with an Medical Research Council (MRC) score ≤ 3 in 53% of patients, however, foot plantar flexion was also impaired in 78% of individuals, with an MRC score of ≤ 3 in 33%. Notably, 77% of patients had a similar degree of weakness of dorsiflexion and plantar flexion (difference of MRC score ≤ 1). Weakness was asymmetric in 22% of individuals. Deep tendon reflexes in the upper limbs and knees were frequently retained or even increased, while ankle reflexes were more typically reduced or absent. Sensation was usually normal in the upper limbs, while sensation to pinprick and vibration in the lower limbs were reduced in 28% and 40% of the cases, respectively.

### Neurophysiology and other investigations

Nerve conduction studies (NCS) were available for review in 109 patients. NCS were from multiple labs with diverse normative values. The overall picture is a motor predominant axonal neuropathy, most evident in the legs (Table 3). This is best illustrated by the finding that the mean amplitude of the tibial nerve was reduced (*x̄* = 2.3 mV), whereas the mean amplitude of the sural nerve was normal (*x̄* = 10 μV). Our data show, however, that upper limb involvement can be observed in approximately half of the cases, reflected by reduced CMAPs amplitude of ulnar (50%) or median (44%) nerves. Motor conduction velocities were abnormally reduced in ∼one-fourth of the cases in the upper and lower limbs, mostly falling into the intermediate range (35–45 m/s). Interestingly, sensory conductions in the upper limbs appeared more frequently affected than in the lower limbs. Reduced SNAP amplitude or slower conduction velocities were observed in up to 76% of cases in the upper limbs but only 27% in the lower limbs, thus confirming the observation of a previous smaller case series.^10^

**Table 3:** Electrophysiological findings

Value	*n*	Mean	SD	Median	Range	Abnormal
Age at baseline, years	89	30.8	14.3	29	12–68	–
**Sensory NCS**						
Median_nerve_SNAP (>15)^a^, μV	98	11.8	10.1	8.2	0–51	**55** (56.1%)
Median_nerve_SCV (>40)^a^, m/s	85	47.6	6.5	48.6	27–69.8	8 (9.4%)
Ulnar_nerve_SNAP (>15)^a^, μV	89	10.3	9.4	7.8	0–47	**68** (76.4%)
Ulnar_nerve_SCV (>40)^a^, m/s	76	48.1	7.7	48.8	29–65.8	10 (13.2%)
Radial_nerve_SNAP (>15)^a^, μV	53	14.2	8.0	13.0	0–43.4	**33** (62.2%)
Radial_nerve_SCV (>40)^a^, m/s	47	53.6	8.0	53.8	35–71.0	3 (6.3%)
Sural_nerve_SNAP (>5)^a^, μV	109	10.7	9.2	10.0	0–56.6	**29** (26.6%)
Sural_nerve_SCV (>40)^a^, m/s	92	43.01	7.3	44.0	26–61	22 (24%)
**Motor NCS**						
Distal_median_nerve_CMAP (>8)^a^, mV	105	8.3	8.5	**7**.**0**	0–54.7	**46** (**43.8%)**
Distal_median_nerve_CV (>45)^a^, m/s	99	49.4	8.9	50.8	31–66.2	**31** (31.3%)
Distal_ulnar_nerve_CMAP (>8)^a^, mV	100	8.3	8.1	**6**.**2**	0.5–57.7	**50** (**50%)**
Distal_ulnar_nerve_CV (>45)^a^, m/s	95	49.9	8.1	50.4	32–67.1	**28** (29,5%)
Distal_peroneal_nerve_CMAP (>5)^a^, mV	104	**2**.**3**	5.2	**0**.**8**	0–39.8	**78** (**75%)**
Distal_peroneal_nerve_CV (>39)^a^, m/s	68	39.7	6.4	41.0	18–51.4	19 (27.9%)
Distal_tibial_nerve_CMAP (>8)^a^, mV	103	**2**.**3**	2.8	**1**.**2**	0–13.2	**75** (**72.8%)**
Distal_tibial_nerve_CV (>38)^a^, m/s	76	40.1	6.6	40.2	22–58.5	20 (26.3%)

Peroneal CMAP is measured from extensor digitorum brevis (EDB). Number of individuals = 109, disease duration = 13.8 ± 12.8 years since the onset of walking difficulties. CMAP = compound motor action potential; CV = conduction velocity; NCS = nerve conduction studies; SCV = sensory nerve conduction velocity; SD = standard deviation; SNAP = sensory nerve action potential.

^a^Note that normative values can vary between labs.

Spine MRI was performed in 27 patients, showing degenerative spine disease in two patients and disc herniation in four cases without evidence of spinal cord compression. Brain MRI was performed in 22 patients showing changes in keeping with the known diagnosis of multiple sclerosis (*n* = 1), probable previous lacunar infarct (*n* = 1) and non-specific white matter changes (*n* = 3).

### Disease severity and progression

Baseline CMTES (*n* = 106) was 6.09 ± 3.7 (0.00–18.0). The neuropathy was considered mild (CMTES 0 to 7) in 77 (72%), moderate (CMTES 8 to 16) in 28 (26%), and severe (CMTES 17 to 28) in one individual. However, it is known that CMTES, which is a compound score weighting sensory and motor impairment in CMT, can underestimate severity in purely or predominantly motor CMT, due to the low scores of items assessing sensory deficit. We assessed the cross-sectional MRC score of first dorsal interosseous (FDI), foot dorsiflexion and foot plantar flexion, as well as CMTES as surrogate markers of disease progression and assessed the association with age, sex and mutation type (nonsense versus missense) (Table 4). Foot dorsiflexion and plantar flexion decreased significantly with age (*P <* 0.001), CMTES increased significantly with age (*P =* 0.003), while FDI did not show significant changes. There was a significant association between sex and foot plantar flexion, with males being more affected. We did not observe a correlation between sex and FDI, foot dorsiflexion, or CMTES. Mutation type (missense versus nonsense) showed no association with CMTES or strength of the muscle group tested. Sixty-seven cases have had a second and most recent examination after 6.9 ± 7.4 years. Foot dorsal and plantar flexion strength declined significantly (*P =* 0.0013 and *P =* 0.001, respectively), while there was a borderline significant decrease of first dorsal interosseous strength (Supplementary Table 1).

**Table 4:** Impact of age, sex and mutation type on disease severity and progression

	Coefficient	Standard error	*t*	*P*-value
**FDI**				
Age	−0.00	0.00	0	1.0
Female gender	−0.03	0.16	−0.22	0.83
Mutation type (missense versus nonsense)	−0.23	0.19	−1.19	0.24
**Foot dorsiflexion**				
Age	−0.03	0.00	−4.2	**<0**.**001**
Female gender	0.41	0.25	1.6	0.11
Mutation type (missense versus nonsense)	−0.14	0.30	−0.46	0.65
**Foot plantar flexion**				
Age	−0.04	0.00	−4.9	**<0**.**001**
Female gender	0.52	0.23	2.3	**0**.**026**
Mutation type (missense versus nonsense)	−0.19	0.28	−0.68	0.50
**CMTES**				
Age	0.08	0.03	3.1	**0**.**003**
Female gender	−1.0	0.79	−1.3	0.21
Mutation type (missense versus nonsense)	1.4	0.84	1.6	0.11
**Change of FDI**				
Disease duration (years)	0.00	0.01	0.84	0.40
Female gender	0.16	0.14	1.1	0.26
Mutation type (missense versus nonsense)	−0.30	0.20	−1.5	0.15
FDI at baseline	0.09	0.10	0.87	0.39
**Change of foot dorsiflexion**				
Disease duration (years)	0.05	0.02	2.4	**0**.**019**
Female gender	−0.74	0.30	−2.5	**0**.**017**
Mutation type (missense versus nonsense)	−0.59	0.10	−1.5	0.14
Foot dorsiflexion at baseline	0.24	0.12	2.0	**0**.**046**
**Change of foot plantar flexion**				
Disease duration (years)	0.02	0.02	0.97	0.34
Female gender	−0.23	0.26	−0.9	0.37
Mutation type (missense versus nonsense)	−0.19	0.34	−0.58	0.56
Foot plantar flexion at baseline	0.11	0.11	1.06	0.29

CMTES = Charcot-Marie-Tooth Examination Score; FDI = first dorsal interosseus muscle.

Finally, we tested the association of change of FDI, foot dorsiflexion and foot plantar flexion with disease duration (time from first examination to most recent examination), mutation type and baseline MRC score of the respective muscle group (Table 3). Foot dorsiflexion decreased significantly by ∼5% per year of disease duration (*P =* 0.017). Males showed a significantly larger change than females (*P =* 0.019), while a low baseline score of foot dorsiflexion was associated with smaller changes over time (*P =* 0.046), likely due to a ceiling effect and difficulties in scoring severely affected muscle groups. There was no association between change of FDI or foot plantar flexion and any of the covariates tested.

## Discussion

In this study, we characterized the genotype and phenotype spectrum of CMT-SORD. The p.Ala253GlnfsTer27 allele is by far the most frequent allele, but other biallelic mutations can cause CMT-SORD. The reported variants cover the entire protein, with moderate clustering in exon 4 and across the co-enzyme binding domain. A loss of enzyme function can be caused by frameshift, truncating, splice, but also by missense mutations in the *SORD* gene. Importantly, elevated serum sorbitol levels provide a valid and reproducible confirmatory test for variants of uncertain pathogenicity.

With an estimated prevalence of 1:2500, hereditary neuropathies are one of the most frequent inherited diseases. Whereas a genetic cause can usually be identified for people with a demyelinating form of CMT, there is a diagnostic gap of ∼50% for CMT patients with axonal neuropathy. Based on high allele frequencies, we postulate that, to date, CMT-SORD is the most common autosomal recessive axonal neuropathy, and our present data confirm that the disease has been diagnosed in many different populations. One challenge is the paralogous pseudogene *SORDP2*, which contains the most frequent pathogenic p.Ala253GlnfsTer27 mutation in 95% of chromosomes. This homologous sequence potentially interferes with genetic testing of this locus and is likely a reason why CMT-SORD was not discovered until 2020.^1^ This molecular testing challenge also applies to Sanger sequencing, where (nested) primers need to be specifically designed to resolve this locus.

Our results represent the first cross-sectional observational study on CMT-SORD and benefited from a global network of expert neurologists who collected detailed information on symptoms, clinical examinations, nerve conduction and mutation status. One limitation is the high number of involved centres and the retrospective design of this study, which may lead to missing data and potential examiner bias. Also, several patients had a neurological comorbidity, which may contribute to their overall disability. The aim of this study was, however, to depict a global real-life cohort representing the full phenotype spectrum of CMT-SORD rather than a more homogeneous and pre-selected cohort, as would be needed for a clinical trial. Of note, we also successfully identified patients originating from Morocco, Brazil, Serbia and Israel—regions that are usually under-represented in CMT clinical research studies.^20,21^ As for other motor predominant CMT2 and dHMN subtypes, the similar involvement of foot dorsiflexion and plantar flexion may represent a clue to suspect the disease. Upper limb and patellar reflexes may be retained or even brisk, suggesting the presence of a possible subclinical involvement of the CNS, as also observed in other axonal CMT subtypes,^22^ and in the absence of relevant changes of imaging.

We use the previously validated CMTES severity scale as surrogate measure to inform on disease progression. In a cross-sectional analysis, foot dorsiflexion, plantar flexion and the CMTES were significantly associated with the age of the subject, and in a subgroup of patients with two separate neurologic evaluations, foot dorsiflexion showed the largest change over time (5 ± 2% per year). The change in foot dorsiflexion was greater in patients with preserved muscle strength at first evaluation, possibly due to a floor effect in more advanced cases. Prospective natural history studies similar to what has been done for other forms of CMT subtypes, including CMT1A,^23^ and CMT due to MPZ,^24^ MFN2,^25^ GJB1^26^ and SH3TC2^27^ mutations, will be needed to accurately track the disease progression and confirm these cross-sectional changes. Biomarkers of neuropathy (e.g. plasma levels of neurofilament light; NFL) and of disease progression (intramuscular fat fraction) will be critical for enabling clinical trials, especially in rare CMT subtypes.^28-30^

We identified a possible effect of male sex on disease severity, which is unusual for recessive diseases. Male sex was significantly associated with the severity of distal lower limb weakness (plantar flexion) and a larger change over time (dorsiflexion). This was paralleled by the higher number of male patients enrolled in the study, who received molecular confirmation of CMT-SORD, with a male to female ratio of 2:1. Interestingly, SORD expression was shown to be androgen-regulated in the human prostate and a putative androgen-responsive regulatory region at the *SORD* promoter has been identified.^31^ We hypothesize that this sex-specific difference may be due to a reduced severity of the neuropathy in females. Finally, a recent study on a *SORD* knockout rat model observed and discussed earlier onset and more severe disease in male animals.^32^ Larger studies will be needed to confirm these observations.

The pathophysiology of CMT-SORD is still unclear. The lack of SORD leads to elevated intracellular sorbitol and serum sorbitol, but how this causes a motor predominant neuropathy is not known. The polyol pathway has long been studied in the context of diabetes and diabetic neuropathy.^14^ Diabetic polyneuropathy is sensory predominant at least initially, and sorbitol is only mildly increased in diabetic patients, later in life. In contrast, sorbitol in inherited SORD deficiency is elevated >10-fold throughout life. This might explain the different modes of peripheral nerve damage: small fibres in diabetic neuropathy and large fibres in CMT-SORD.

In summary, we have described the largest cohort of CMT-SORD patients to date. CMT-SORD is a motor-predominant, recessive CMT/dHMN with mild-to-moderate severity. We recommend that *SORD* be included in all genetic screens for inherited neuropathy, and that sorbitol be measured in serum/plasma or, as recently shown, in urine,^33^ in patients carrying yet unseen or unclassified *SORD* changes. Diagnosing CMT-SORD will become imperative if disease-modifying therapies are found. This study will have immediate translational value for diagnoses and treatment efforts for CMT patients.

## Supplementary Material

awaf021_Supplementary_Data

## Data Availability

Data are not publicly available due to lack of patients’ consent. De-identified data are available upon reasonable request to the corresponding author.
