# Supplementary material for: Genotype and phenotype spectrum of Charcot-Marie-Tooth disease due to mutations in SORD
Source: Brain. 2025 Feb 13;148(10):3737–47. doi: 10.1093/brain/awaf021 (PMC12493047; doi:10.1093/brain/awaf021)
Supplement: awaf021_Supplementary_Data [file awaf021_supplementary_data.zip › brain-2024-01355-File011.pdf]

Supplementary Table 1. Longitudinal changes of muscle strength in distal upper and lower limb muscles in CMT-SORD patients.

	**Baseline (MRC, mean ± SD)**	**Second examination (MRC, mean ± SD)**	**Change**	***P*-value**
**FDI**	4.36 ± 0.68	4.23 ± 0.81	0.13± 0.52	0.055
**Foot dorsiflexion**	3.06 ± 1.27	2.54 ± 1.53	0.51 ± 1.26	**0.0013**
**Foot plantar flexion**	3.84 ± 1.19	3.43 ± 1.41	0.42 ± 0.97	**0.0010**

FDI = first dorsal interosseus muscle. Number of individuals =67, follow-up time = 6.9 ± 7.4 years
