# Supplementary figures and images for: Genotype and phenotype spectrum of Charcot-Marie-Tooth disease due to mutations in SORD
Source: Brain. 2025 Feb 13;148(10):3737–47. doi: 10.1093/brain/awaf021 (PMC12493047; doi:10.1093/brain/awaf021)

Supplementary Figure 1. Fasting and post-prandial serum sorbitol level in CMT-SORD.

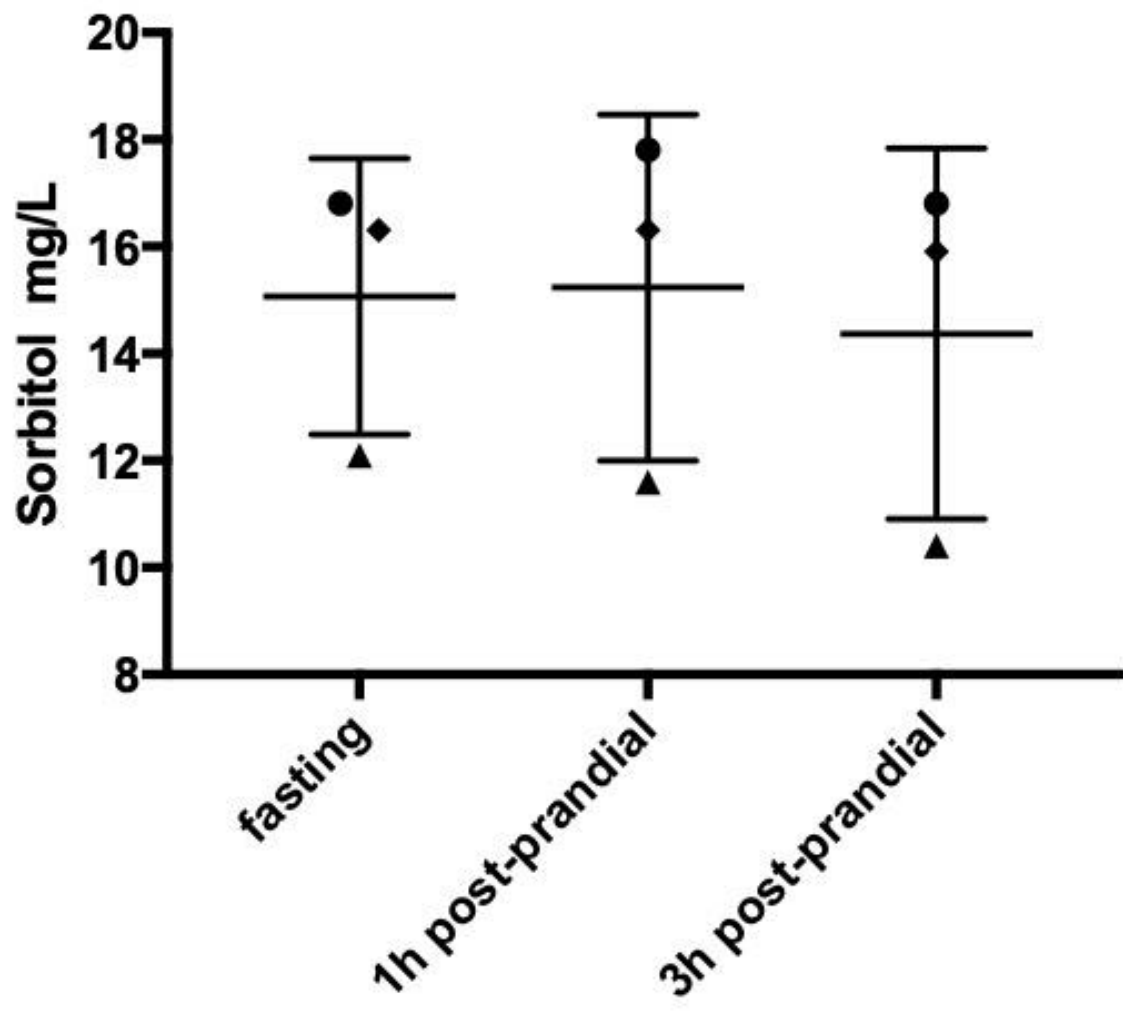

Supplement: awaf021_Supplementary_Data [file awaf021_supplementary_data.zip › brain-2024-01355-File010.pdf]
